# The effects of football practice on children's fundamental movement skills: A systematic review and meta-analysis

**DOI:** 10.3389/fped.2022.1019150

**Published:** 2022-12-20

**Authors:** Xiaojin Mao, Jingyue Zhang, Yulian Li, Yuang Cao, Meng Ding, Weidong Li, Lixia Fan

**Affiliations:** ^1^ Department of Physical Education, Shandong Normal University, Jinan, China; ^2^ Education and Sports Bureau of Huaiyin District, Jinan, China

**Keywords:** children, soccer, physical practice, fundamental movement skills, meta-analysis

## Abstract

The purpose of this systematic review and meta-analysis was to explore the effects of different soccer practices on fundamental movement skills (FMS) of children of different ages and genders, in order to help children to improve their fundamental movement skills through soccer practice more effectively. The databases of CNKI, Wanfang database, Pubmed, Web of science and Cochrane library were searched to collect relevant studies on the effects of soccer practices on FMS, and the quality of the included studies was evaluated by using the Cochrane Risk of Bias Tool, and Meta-analysis was conducted by Review Manager 5.4 software. 16 studies were finally included, with a total of 3,121 subjects were included. The results showed that soccer had a positive effect on linear sprint ability [SMD 95% CI = −0.37 (−0.61, −0.14), *P* = 0.002], horizontal jump [SMD 95% CI = 0.22 (−0.34, 0.77), *P* = 0.003], object control [SMD 95% CI = 1.32 (0.8, 1.85), *P* = 0.0003], Closed-eye single-leg test [SMD 95% CI = 0.87(0.48,1.25), *P* < 0.0001],while countermovement jump [SMD 95% CI = 0.50(−0.35,1.35), *P* = 0.25] and flamingo balance [SMD 95% CI = −0.16(−0.31,−0.02), *P* = 0.03] had a less significant effect. Meanwhile, the effect of the practice was mainly influenced by the total duration of the intervention, age and gender of the intervention subjects, in which the total intervention time longer than 1,800 min promoted linear sprint ability, horizontal jump and flamingo balance test better than those below 1,800 min; the promotion effect of linear sprint ability and horizontal jump was better in children aged 7–9 years than 10–13 years, while Children aged 10–13 years showed better improvement in Closed-eye single-leg test after the intervention than 7–9 years; Girls were better promoted in linear sprint ability and Closed-eye single-leg test, but the horizontal jump is better for boys to improve the effect. It is recommended that the effects of different soccer practice contents on fundamental movement skills can be further explored in the future to improve the relevance and efficiency of fundamental movement skill development for children.

## Introduction

Fundamental movement skills (FMS) refers to the ability to coordinate the use of human basic movements (1), and is an important part of motor skills, consisting of locomotion, such as walking, running, jumping, sliding, etc.; object control, such as grasping, throwing, kicking, catching, etc; stability, such as rotation, turning, bending, etc (2), are considered to be the basic “building blocks” of advanced and complex motor sequences required for various sports, games, or physical activities (3). Childhood is an important stage of motor development, and the level of gross and fine motor movements at this stage has a significant impact on future motor development (4, 5), and failure to acquire FMS at the appropriate age may increase the risk of children experiencing long-term physical and mental health problems (6) However, at a time when physical inactivity and sedentariness in young children are global problems (7), increasing numbers of children have severely limited development of fundamental movement skills (8). The implementation of effective exercise interventions to improve children's FMS is necessary in this current situation, but current studies have focused on the effects of exercise interventions on children's physical fitness. Studies have shown that regular participation in physical activity is beneficial in improving physical fitness (9), and considering that FMS can indicate levels of physical fitness, therefore, there may be a correlation between FMS and physical activities (10, 11), so that soccer practice may be a viable way to develop FMS in children. Some studies have confirmed that soccer practices promote overall FMS in children, such as P. F. Nazario (12) in 2013, who confirmed the effect of soccer on children's locomotion such as linear sprint ability, sliding, horizontal jump, and object control such as catching, throwing, and kicking through a 25-week soccer practices, and Yangyang Guo (13) in 2020, who came to a similar conclusion through an 8-week soccer intervention. But there were some differences between the two studies in the effects of other parts of object control and locomotion. In addition, there are differences in the effects of soccer on children's balance (14–20), And the content of the practice and the characteristics of the subjects may be the reasons for the different results.

Previously, some reviews have been conducted for the relationship between physical activity and FMS (21–24), but the experimental subjects were mostly young children (21, 22) or young children and children and adolescents as a whole (23, 24), and fewer review have been conducted for children. It has been demonstrated that physical activity has a facilitative effect on FMS in young children aged 2–6 years (21, 22) and that moderate and high motor intensity physical exercises have a low and moderate relationship with some FMS subordinate skills (22). And another review (23) conducted a study on resistance exercise and FMS in children and adolescents aged 5–18 years and found that resistance training had a facilitative effect on FMS such as running, jumping and throwing. Specifically for soccer, some researchers (25) have conducted review for the effects of different training methods on the physical fitness of athletes, but no studies have been conducted for different soccer practices on different groups of children with FMS.

Therefore, this study considers the following deficiencies in the current domestic and international studies of soccer practices for FMS: (1) The effects of different soccer practice times and methods on children's FMS are unclear. (2) The effects of soccer practices on FMS subordinate skills are controversial. (3) The effects of interventions for different age and gender groups of children are unclear.

## Materials and methods

### Experimental approach to the problem

This study was guided by the Preferred Reporting Items for Systematic Evaluation and Meta-Analysis (PRISMA) (26) and registered in the PROSPERO database under number CRD42022340727. By searching databases such as CNKI, Wanfang database, Pubmed, Web of science, and Cochrane library, the search time period is from the creation of the database to April 2022, with the last search date being April 27, 2022. The search formula in Chinese and English was (FMS OR fundamental movement skill OR basic motor skill OR gross motor skill OR physical fitness OR gross motor skill OR speed OR run OR jump OR object control OR stability OR balance) AND (children OR kid OR student) AND (football OR soccer).

### Eligibility criteria

#### Inclusion criteria

(1) Randomized controlled trials about soccer practices for children with FMS or subordinate skills of FMS, with language limited to Chinese and English. (2) Normally physically developed children aged 7–13 years, regardless of gender. (3) The total length of soccer practice is over 8 weeks.

#### Exclusion criteria

(1) Study that did not satisfy the inclusion criteria. (2) Children with behavioral disorders. (3) Unreasonable interventions in the experimental and control groups, such as additional psychological interventions. (4) Lack of basic information about the experimental subjects. (5) Duplicate detections of the study. (6) Systematic analysis of the study. (7) Study lacking full text.

#### Interventions

The experimental group used soccer practices (The total length of soccer practice >8 weeks, intensity, frequency, and form of practice were not limited); the control group did not have additional intervention or participated in the physical education course normally.

#### Outcome indicators

(1) linear sprint ability, linear sprint ability is one of the locomotion, this study used 15 m–50 m fast running as the test method in s; (2) jumping ability, jumping ability is also one of the locomotion, this study used horizontal jump and countermovement jump as the test method; (3) object control, through TGMD-2 as the test method, mainly including two-hand striking a stationary ball, stationary dribbling, catching, kicking, overhand throwing and underhand rolling (27); (4) balance, which was analyzed by two test methods, closed-eye single-leg test and flamingo balance (28).

### Data synthesis and analysis

In this study, two researchers conducted the quality evaluation and data extraction, a third researcher was involved in the joint work for the areas of disagreement. After extraction, the difference between the pre- and post-intervention means and the standard deviation was calculated by subtracting the base-measure value from the post-measure value, and the standard deviation was calculated by “SD difference ^2^ = SD _base value_^2^ + SD _post value_^2^ − 2*R*SD _base value_ * SD _post value_, *R* = 0.5″ (29). According to the Cochrane Handbook of Systematic Evaluation (30), the baseline is balanced and comparable between groups in randomized controlled trials, and theoretically there is no difference between the comparison of post-intervention measurements and the comparison of pre- and post-intervention differences. In this study, post-measure values of object control indicators were analyzed for comparison because of the relatively small number of study and missing baseline for some indicators.

### Quality evaluation and statistical analyses

**Figure 1:**
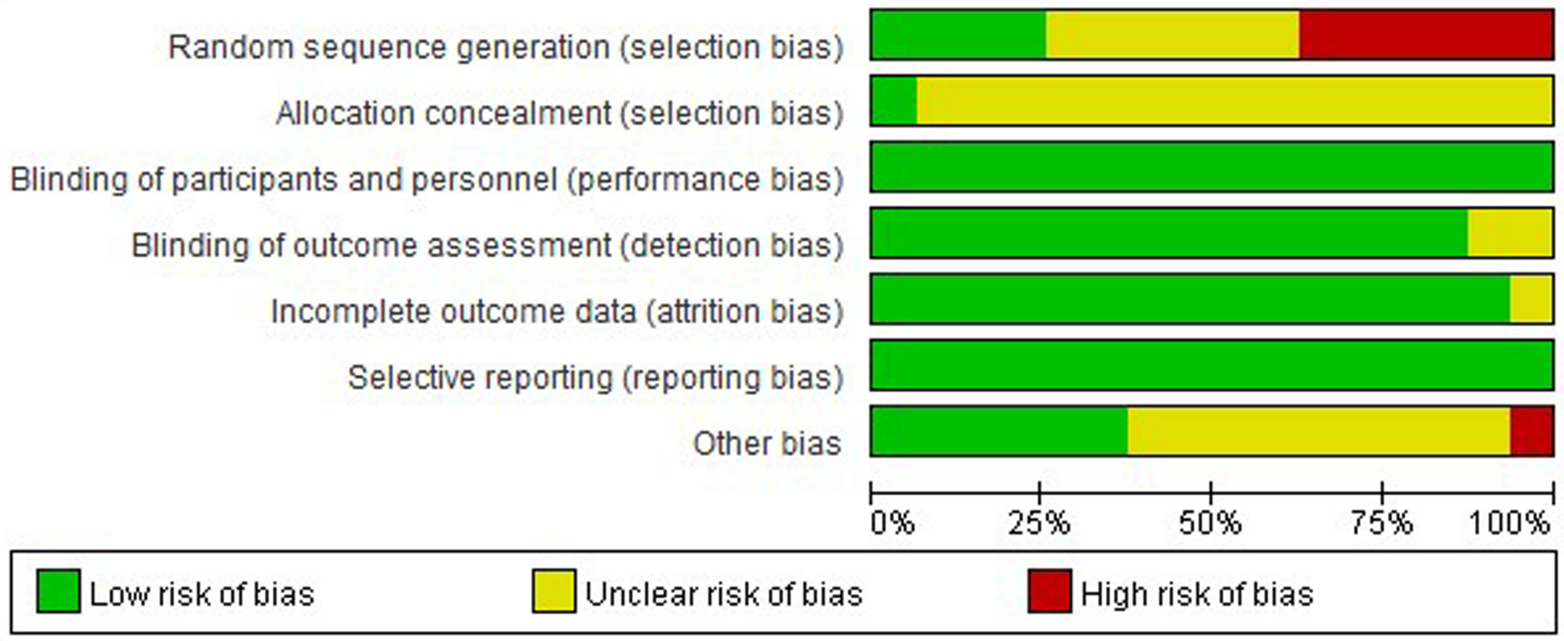
Risk of bias summary for all included study.

**Figure 2:**
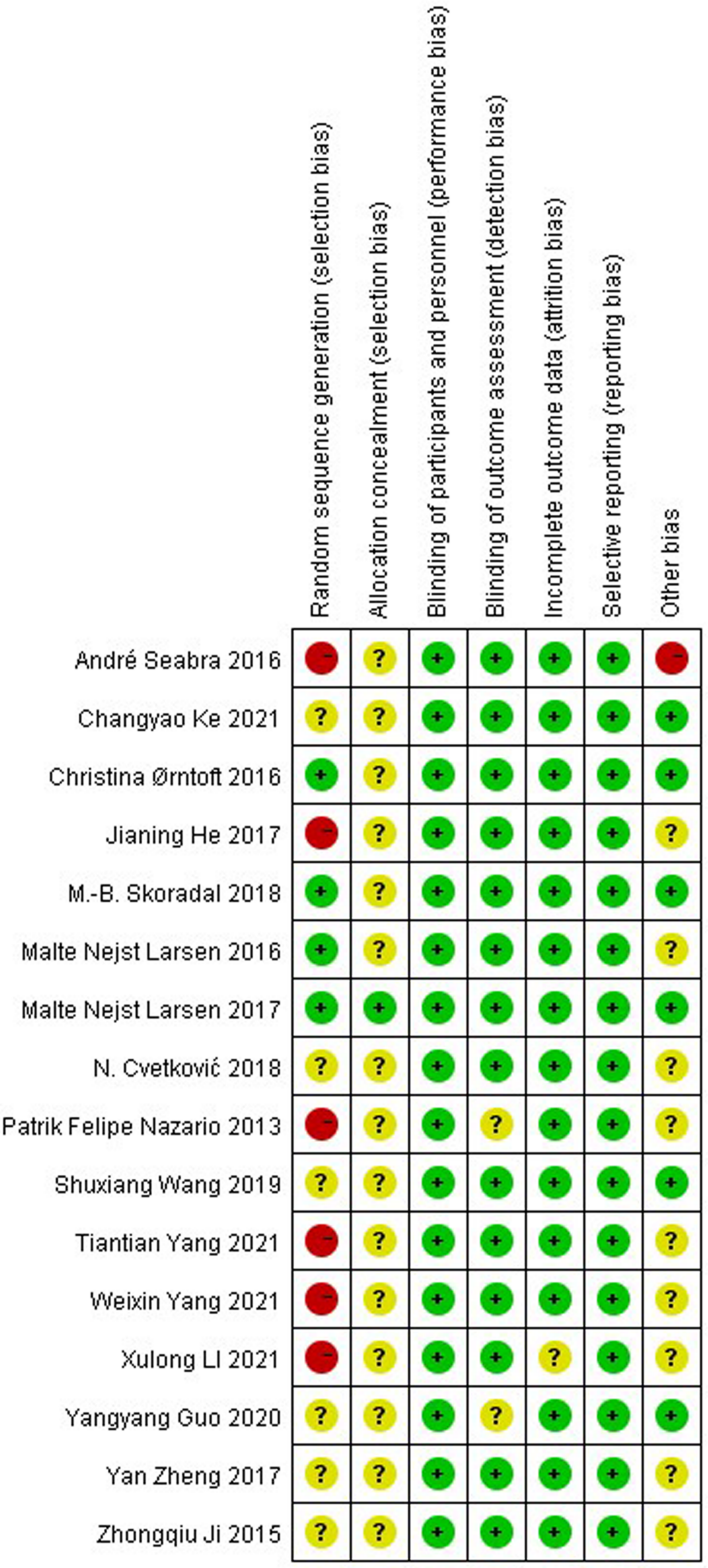
Risk of bias graph of the included studies.

Cochrane Handbook for Systematic Reviews of Interventions (30) were used to score the included studies for (1) random sequence generation; (2) allocation concealment; (3) blinding of participants and personnel; (4) blinding of outcome assessment; (5) incompleteness of outcome data; (6) selective reporting; (7) other bias. Each criterion has three options of low risk, high risk, and unclear risk, and can be classified in category A when the number of low risks in the study is ≥4, in category B when the number of low risks in the study is ≥2 and <4, and in category C when the number of low risks in the study is <2. As shown in Figures 1, 2, only 4 studies were standardized in the generation of random sequences generation (14–16, 31), and most of the studies were not assigned to concealment, because the sports interventions could not be set up double-blind, most of the experiments were mainly instrumental measurements, and the subjective influence of the evaluator on the experimental results was small, Therefore, “blinding of outcome assessment” scores are usually high. A total of 14 included studies met the criteria of category A (13–18, 20, 31–37) and 2 met the criteria of category B (12, 19), and the overall quality of the study was high.

Meta-analysis was performed using Review Manager 5.4 software (38). Heterogeneity was tested using *I*^2^, and heterogeneity was considered to exist when *I*^2^ > 50% (39), and a random-effects model was selected; while *I*^2^ < 50% was used to study homogeneous effects and a fixed-effects model was selected. Standardized mean difference (SMD) and 95% confidence interval (95% CI) were chosen as the effect scale to combine effect sizes, and small effects were considered when the absolute value of SMD was 0–0.4, moderate effects were considered when 0.4–0.8, and high effects were considered when greater than 0.8, and differences were considered significant when *P* < 0.05, and subgroup analysis was performed for highly heterogeneous study results (39).

## Results

### Study search results

**Figure 3:**
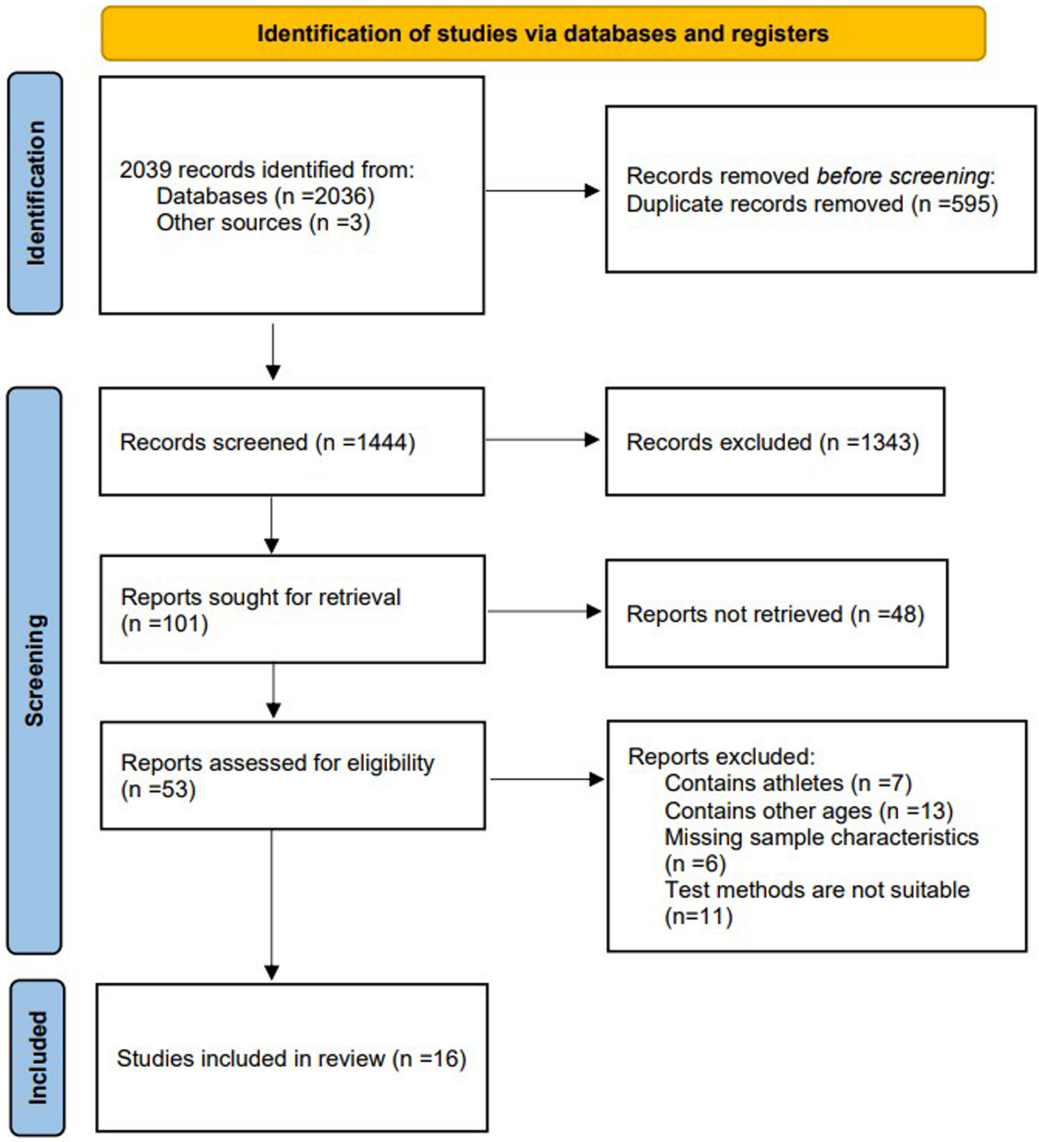
Flow diagram of the selected studies.

Through the search of Chinese and English study, a total of 2,036 studies were retrieved, 595 duplicate studies were excluded, 1,391 studies were excluded after reading the titles and abstracts, 37 studies were excluded by reading the full text, and 16 studies were finally screened (12–20, 31–37), including a total of 7 studies in English (12, 14–16, 31–33) and 9 studies in Chinese (13, 17–20, 34–37) (Figure 3).

### Study characteristics

In all included studies(Tables 1, 2), there were 3,121 children aged 7–13 years, including 1,958 in the experimental group and 1,163 in the control group; there were 7 studies with more than 100 participants (14–16, 20, 31, 34, 35), 8 studies with 20–100 participants (12, 13, 17–19, 33, 36, 37), and 1 study with less than 20 participants (32); 5 studies interventions were only for boys (13, 15, 17, 32, 33), 7 studies were co-educational interventions (14, 19, 20, 31, 34–36), and 4 did not specify the proportion of interventions (12, 16, 18, 37). The interventions were all based on soccer practice and 4 studies stated the intensity of the intervention (15, 19, 36, 37); a total of 10 studies (13, 14, 17, 18, 20, 31, 33–36) for less than 20 weeks and 6 studies (12, 15, 16, 19, 32, 37) for more than 20 weeks; a total of 11 studies practiced at least 3 times per week (13, 15–19, 32–36) and 5 less than 3 times (12, 14, 20, 31, 37); 6 studies with weekly intervention time less than 120 min (13–15, 18, 20, 31) and 10 with intervention time more than 120 min (12, 16, 17, 19, 32–37); a total of 15 studies with baseline testing and comparison before the experiment (13–20, 31–37); for test results, a total of 13 studies with less association with rater subjectivity, such as horizontal jump, linear sprint, and Closed-eye single-leg test (14–20, 31–37), and a total of 2 studies with rater subjectivity scoring (12, 13); all included studies were published after 2013.

**Table 1:** Characteristics of the experimental subjects included in the study.

Number	Author	Year		Experimental subjects	
			Crowd	Age (years)	Experimental group (male: female)/Control group (male: female)
1	A. Seabra (32)	2016	NC	8–12	9 (9 : 0)/8 (8 : 0)
2	C. Ørntoft (14)	2016	NC	10–12	386 (200 : 186)/140 (57 : 83)
3	M.B. Skoradal (31)	2018	NC	11.1 ± 0.3	292 (146 : 146)/100 (50 : 50)
4	M.N. Larsen (15)	2017	NC	10–12	829 (829 : 0)/439 (439 : 0)
5	M.N. Larsen (16)	2016	NC	EG:9.3 ± 0.4 CG:9.3 ± 0.3	91 (Unknown)/108 (Unknown)
6	N. Cvetković (33)	2018	NC	11–13	10 (10 : 0)/14 (14 : 0)
7	P. F. Nazario (12)	2013	NC	9 ± 1.0	16 (Unknown)/25 (Unknown)
8	Shuxiang Wang (17)	2019	NC	EG:9.2 ± 0.2 CG:9.2 ± 0.1	20 (20 : 0)/23 (23 : 0)
9	Yangyang Guo (13)	2020	NC	EG:8.80 ± 0.77 CG:8.85 ± 0.75	20 (20 : 0)/20 (20 : 0)
10	Changyao Ke (34)	2021	NC	10–11	60 (30 : 30)/60 (30 : 30)
11	Weixin Yang (18)	2021	NC	EG:9.37 ± 0.38 CG:9.27 ± 0.28	32 (Unknown)/32 (Unknown)
12	Xulong li (19)	2021	NC	EG:9.23 ± 0.45 CG:9.17 ± 0.45	38 (19 : 19)/38 (10 : 9)
13	Zhongqiu Ji (20)	2015	NC	9–11	58 (28 : 30)/60 (30 : 30)
14	Tiantian Yang (35)	2021	NC	8–9	50 (30 : 20)/50 (30 : 20)
15	Jianing He (36)	2019	NC	10–12	16 (8 : 8)/16 (8 : 8)
16	Yan Zheng (37)	2017	NC	7.13 ± 0.14	31 (Unknown)/30 (Unknown)

Note: NC is normally developing children; EG is the experimental group; CG is the control group.

**Table 2:** Status of interventions included in the study.

Number	Author	Interventions		
		Training content and ratio	Group: Total time/week frequency/sub-hour (week/time/min)	Training intensity
1	A. Seabra (32)	EG: 10–20 min warm-up, 40–60 min technical practice and competition,10 min relaxation CG: Unknown	EG:26 weeks*4 times*60–90_ _min CG:26 weeks	Unknown
2	C. Ørntoft (14)	EG: Warm-up, technical practice, competition CG: Not involved in sports	EG:12 weeks*2 times*45_ _min CG:12 weeks	Unknown
3	M.B. Skoradal (31)	EG: “FIFA 11 for Health”; Regular physical education courses CG: Normal physical education course	EG:13 weeks*2 times*45_ _min; 13 weeks CG:13 weeks	Unknown
4	M.N.Larsen (15)	EG:3v3 soccer training CG: Normal physical education course	EG:43 weeks*5 times*12_ _min CG:43 weeks	70%–90% HRmax
5	M.N. Larsen (16)	EG: 3v3 soccer training CG: Normal physical education course	EG:43 weeks*3 times*40_ _min CG:43 weeks	Unknown
6	N. Cvetković (33)	EG:10 min warm-up, 4*8 min soccer game, 10 min relaxation; normal physical education course CG: Normal physical education course	EG:12 weeks*3 times*60 min; 12 weeks*2 times CG:12 weeks	Unknown
7	P. F. Nazario (12)	EG: Soccer Practice; Physical Education Course CG: Physical Education Course	EG:25 weeks*2 times*90 min;25 weeks*2 times*50_ _min CG: 25 weeks*2 times*50_ _min	Unknown
8	Shuxiang Wang (17)	EG:8 min warm-up, 32 min soccer technique, physical exercise, 5 min relaxation CG: Normal physical education course	EG:12 weeks*3 times*45_ _min CG:12 weeks	Unknown
9	Yangyang Guo (13)	EG: Preparation part, ballistic exercise, ending part CG: Preparation section, fitness exercises, end section	EG:8 weeks*3 times*30_ _min CG: 8 weeks*3 times*30_ _min	Unknown
10	Changyao Ke (34)	EG: physical, technical, tactical, and general application skills training; normal physical education courses CG: Normal Physical Education Course	EG:16 weeks*5 times*45 min; 16 weeks CG:16 weeks	Unknown
11	Weixin Yang (18)	EG: 8 min warm-up, 8 min technical training, 9 min game, 10 min shooting; physical education class CG:7 min preparation activities, basic part, 23 min exercises, 5 min relaxation	EG:12 weeks*3 times*35 min; 12 weeks*1 time*35_ _min CG: 12 weeks*4 times*35_ _min	Unknown
12	Xulong li (19)	EG: Basic soccer skills, special quality CG: Normal physical education courses (no ball games)	EG:20 weeks*5 times*60_ _min CG: 20 weeks*5 times*60_ _min	60%–70% HR_max_
13	Zhongqiu Ji (20)	EG: Inside foot passing is the main focus, supplemented by ballistic exercises and lower body strength exercises; CG: Normal physical education course (no soccer practice)	EG:12 weeks*2 times*40_ _min CG: 12 weeks*2 times*40_ _min	Unknown
14	Tiantian Yang (35)	EG:8 min warm-up, 32 min soccer training. 5 min relaxation stretch. CG:8 min warm-up, 32 min basic exercises The activity is relaxing for 5 min.	EG:16 weeks*3 times*45_ _min CG:16 weeks*3 times*45_ _min	Unknown
15	Jianing He (36)	EG: ball practice, technical practice CG: Normal teaching activities	EG:12 weeks*3 times*90_ _min CG:12 weeks	110–130 HR_x−bar_
16	Yan Zheng (37)	EG:15 min warm-up, 15 min quality training, 60 min soccer technical training, 30 min game; normal sports course CG: Normal physical education course	EG:32 weeks*1 time*120_ _min CG:32 weeks	72.70% HRmax

*Represent multiplication.

## Results of meta-analysis

### Meta-analysis of the effect of soccer on locomotion

#### Linear sprint ability

**Figure 4:**
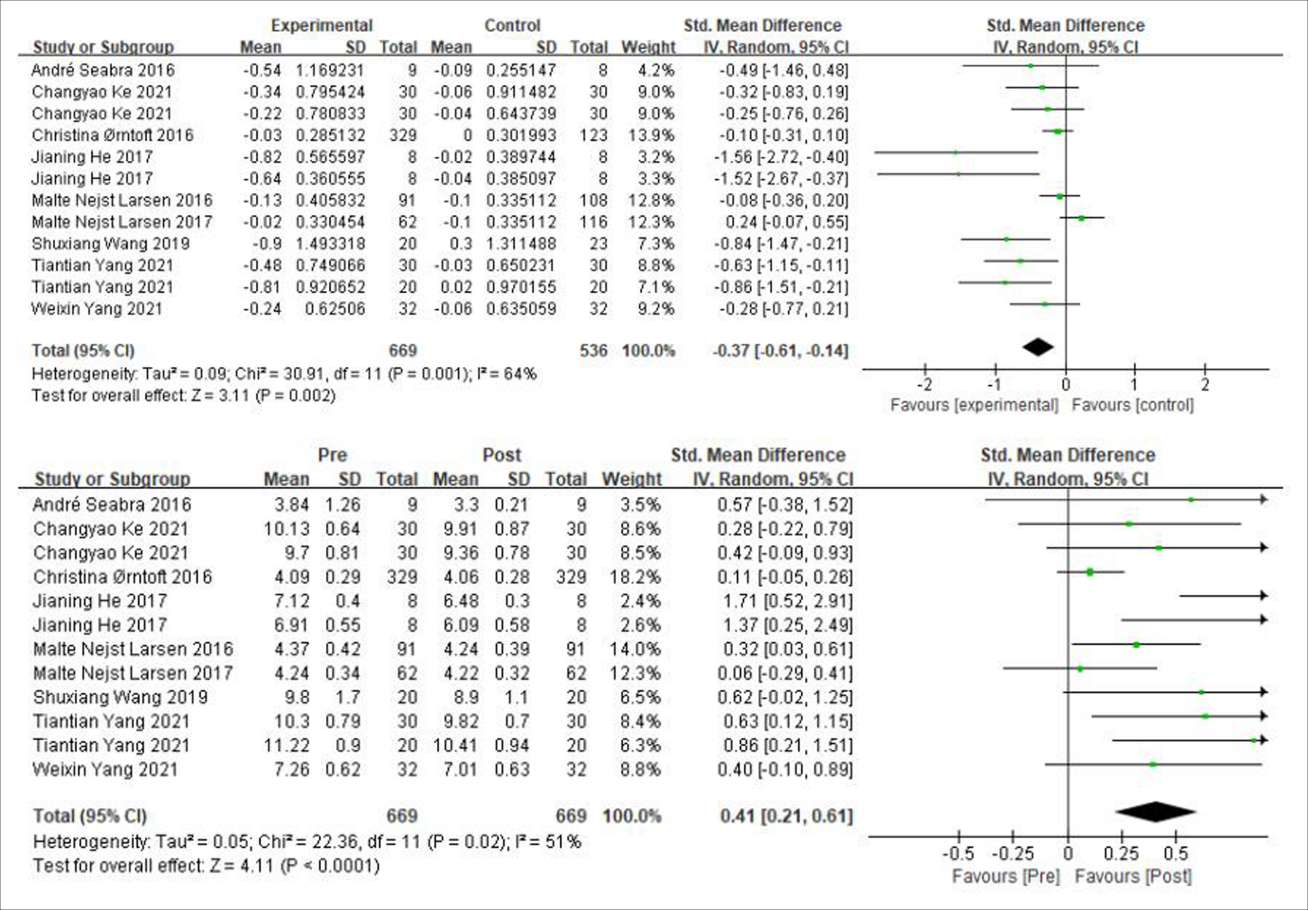
A meta-analysis of the results of linear sprint ability was conducted for comparisons between the control and experimental groups and for comparisons between the experimental groups pre- and post-intervention.

Linear sprint ability is measured in seconds, and a decrease in value represents an increase in ability. A total of 9 papers containing studies on linear sprint ability in soccer. As shown in Figure 4, *χ*² = 30.91, df = 11 (*P* = 0.001), *I*^2^ = 67% between the experimental and control groups, which can be considered as heterogeneity between the two groups, using a random effects model. The results showed that the combined sample size was 1,205 cases, SMD = −0.37, 95% CI: [−0.61, −0.14], Z = 3.11, *P* = 0.002, the combined effect was statistically significant, the small diamond-shaped squares fell to the left of the null line and did not intersect, indicating that there was a moderate effect and the soccer practices had a facilitative effect on children's linear sprint ability. The pre and post intervention in the experimental group *χ*² = 22.36, df = 11 (*P* = 0.02), *I*^2^ = 51%, SMD = 0.41, 95% CI: [0.21, 0.61], *P* < 0.0001, indicating the existence of a medium effect size and an increase in linear sprint ability in the experimental group after the intervention.

#### Jumping ability

**Figure 5:**
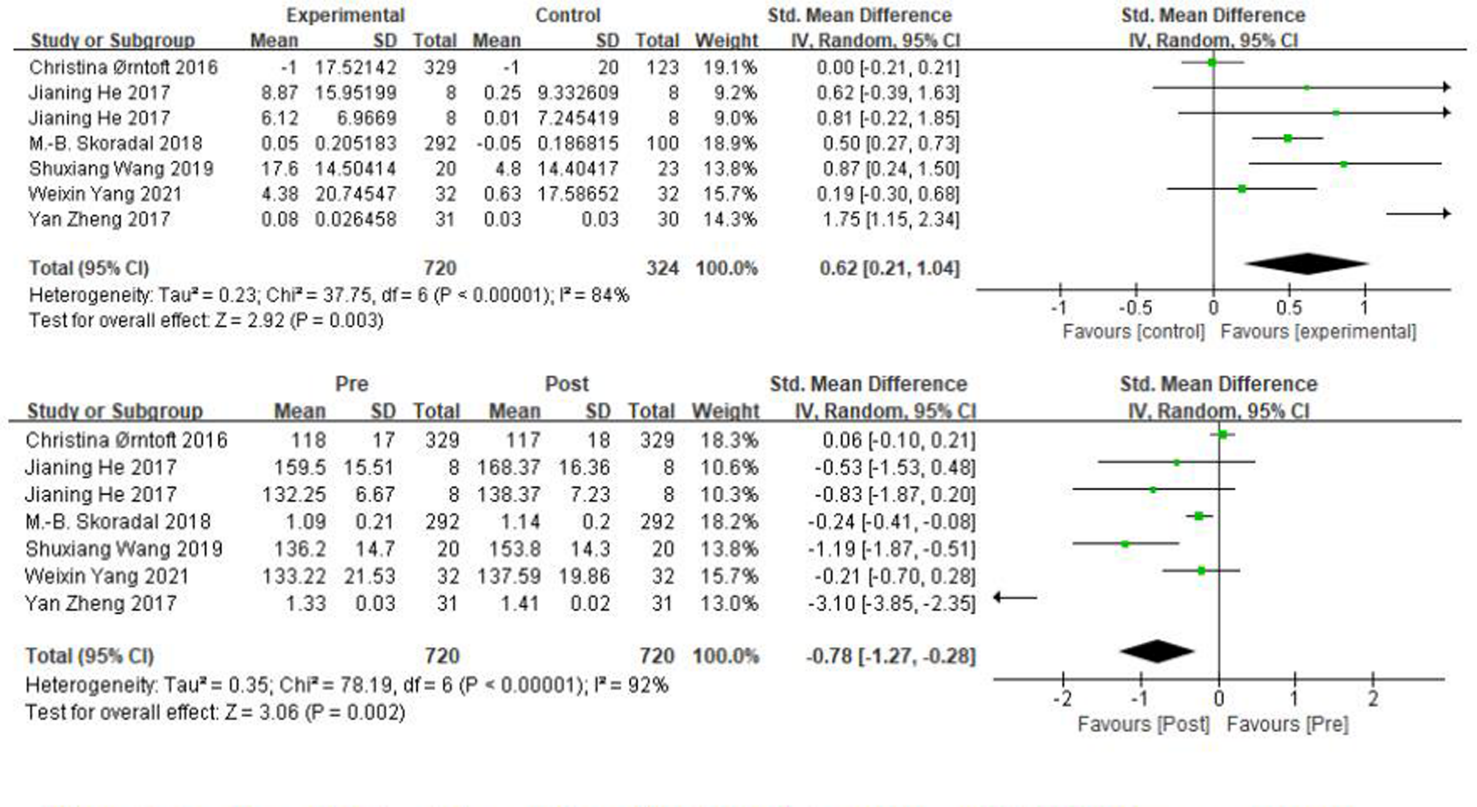
A meta-analysis of the results of horizontal jump was conducted for comparisons between the control and experimental groups and for comparisons between the experimental groups pre- and post-intervention.

A total of 9 studies were included in jumping ability, mainly using horizontal jump and countermovement jump to measure children's jumping ability. In Figure 5, there was a high heterogeneity between the experimental group of horizontal jump and the control group with *χ*² = 37.75, df = 6 (*P* < 0.00001), *I*^2^ = 84%, and a random effects model was used. The results showed that the sample size was 983 cases, the combined effect was statistically significant, SMD = 0.62, 95% CI: [0.21, 1.04], *P* = 0.003, the small diamond-shaped squares were located to the right of the null line and did not intersect, indicating the presence of a moderate effect and a better promotion of the soccer practices on the horizontal jump. Pre and post intervention in the experimental group *χ*² = 78.19, df = 6 (*P* < 0.00001), *I*^2^ = 92%, SMD = −0.78, 95% CI: [−1.27, −0.28], *P* = 0.002, |SMD| > 0.4, indicating the presence of a moderate effect size and a significant increase in the level of horizontal jump after the intervention in the experimental group.

**Figure 6:**
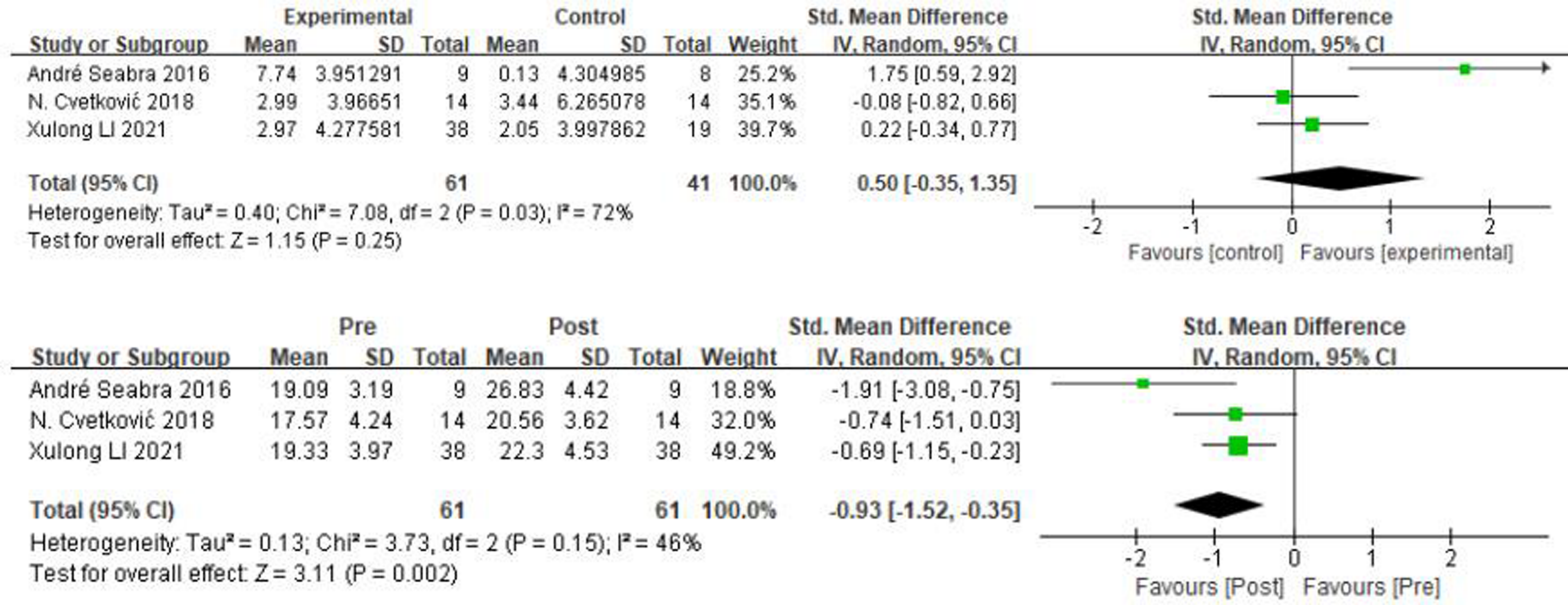
A meta-analysis of the results of countermovement jump was conducted for comparisons between the control and experimental groups and for comparisons between the experimental groups pre- and post-intervention.

From Figure 6, it can be obtained that the countermovement jump *χ*² = 7.08, df = 2 (*P* = 0.03), and *I*^2^ = 72% between the experimental and control groups, which can be considered as heterogeneity between the two groups, and Meta-analysis was performed with a random effects model. The results showed that the sample size was 102 cases, SMD = 0.50, 95% CI: [−0.35, 1.35], *P* = 0.25, and the small diamond-shaped square intersected with the null line, indicating that the difference between the groups was not statistically significant. Pre and post intervention in the experimental group *χ*² = 3.73, df = 2 (*P* = 0.15), *I*^2^ = 46%, SMD = −0.83, 95% CI: [−1.2, −0.45], *P* < 0.0001, |SMD| > 0.8, the small diamond-shaped square was located to the left of the null line and did not compare, indicating a significant change in the countermovement jump before and after the experimental group.

### Meta-analysis of the effect of soccer on object control ability

**Figure 7:**
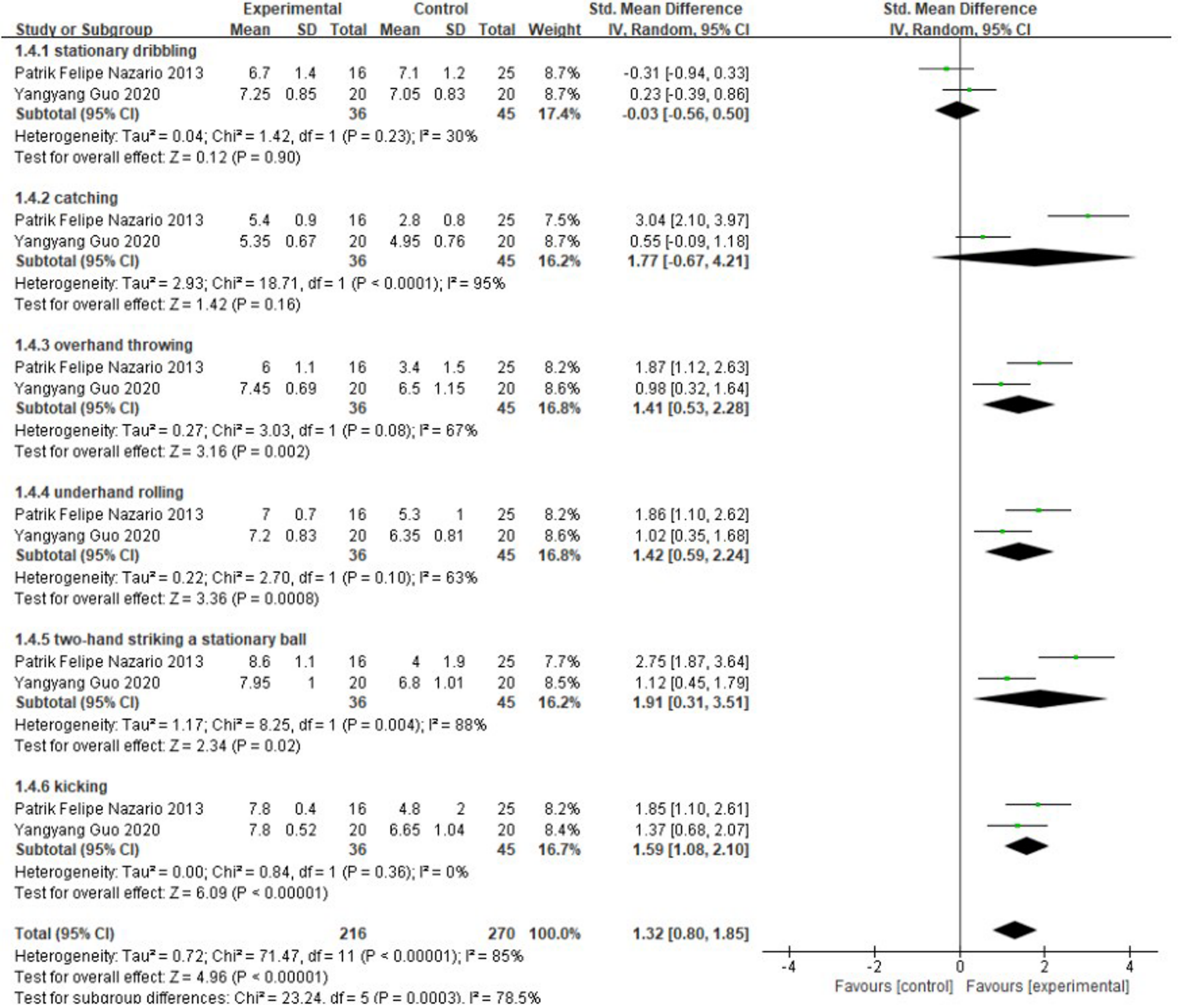
A meta-analysis of the results of object control was conducted for comparisons between the control and experimental groups.

From Figure 7, the effect of soccer on children's object control *χ*² = 71.47, df = 11 (*P* < 0.00001), *I*^2^ = 85%, therefore, Meta-analysis was performed using a random effects model with SMD = 1.32, 95% CI: [0.80, 1.85], *P* < 0.0001, SMD > 0.8, indicating that the soccer intervention had a better facilitation effect on children's object control, and there were significant differences between subgroups.

In stationary dribbling, *χ*² = 1.42, df = 1 (*P* = 0.23), *I*^2^ = 30%, there was homogeneity between the two groups, SMD = −0.03, 95% CI: [−0.56, 0.50], *P* = 0.9, |SMD| < 0.4, and the diamond-shaped small square intersected with the null line, indicating that the soccer sport intervention had no improvement effect on children's stationary dribbling.

In catching ability, *χ*² = 18, 71, df = 1 (*P* = 0.08), *I*^2^ = 95%, there was heterogeneity between the experimental and control groups, SMD = 1.77, 95% CI: [−0.67, 4.21], *P* < 0.0001, SMD > 0.8, and the diamond-shaped cubes intersected the null line, indicating that the soccer sports intervention had no effect on the children's ball catching ability.

There was heterogeneity between the two groups of overhand throwing with *χ*² = 3.03, df = 1 (*P* = 0.08), *I*^2^ = 67%, SMD = 1.41, 95% CI: [0.53, 2.28], *P* = 0.02, SMD > 0.8, and the diamond-shaped cube was located to the right of the null line and did not intersect, indicating that the soccer sport intervention had an improving effect on children's overhand throwing.

In the underhand rolling, *χ*² = 2.7, df = 1 (*P* = 0.1), *I*^2^ = 63%, there was heterogeneity between the experimental and control groups, SMD = 1.42, 95% CI: [0.59, 2.24], *P* = 0.0008, SMD > 0.8, and the small diamond-shaped squares were located to the right of the null line and did not intersect, indicating that the soccer sport intervention had an improving effect on children's underhand rolling.

There was heterogeneity between the two groups of two-hand striking a stationary ball *χ*² = 8.25, df = 1 (*P* = 0.004), *I*^2^ = 88%, SMD = 1.91, 95% CI: [0.31, 3.51], *P* = 0.02, SMD > 0.8, and the small diamond-shaped square was located to the right of the null line and did not intersect, indicating that the soccer practices had an improving effect on children's two-hand striking a stationary ball.

Kicking ability is an important manifestation of lower limb object control ability, *χ*² = 0.84, df = 1 (*P* = 0.36), *I*^2^ = 0%, SMD = 1.59, 95% CI: [1.08, 2.1], *P* < 0.0001, SMD > 0.8 between the two groups, the small diamond-shaped squares were located to the right of the null line and did not intersect, indicating that there was a height effect and that the soccer practices had an effect on children's lower limb object control ability was improved.

### Meta-analysis of the effect of soccer on stabilization ability

**Figure 8:**
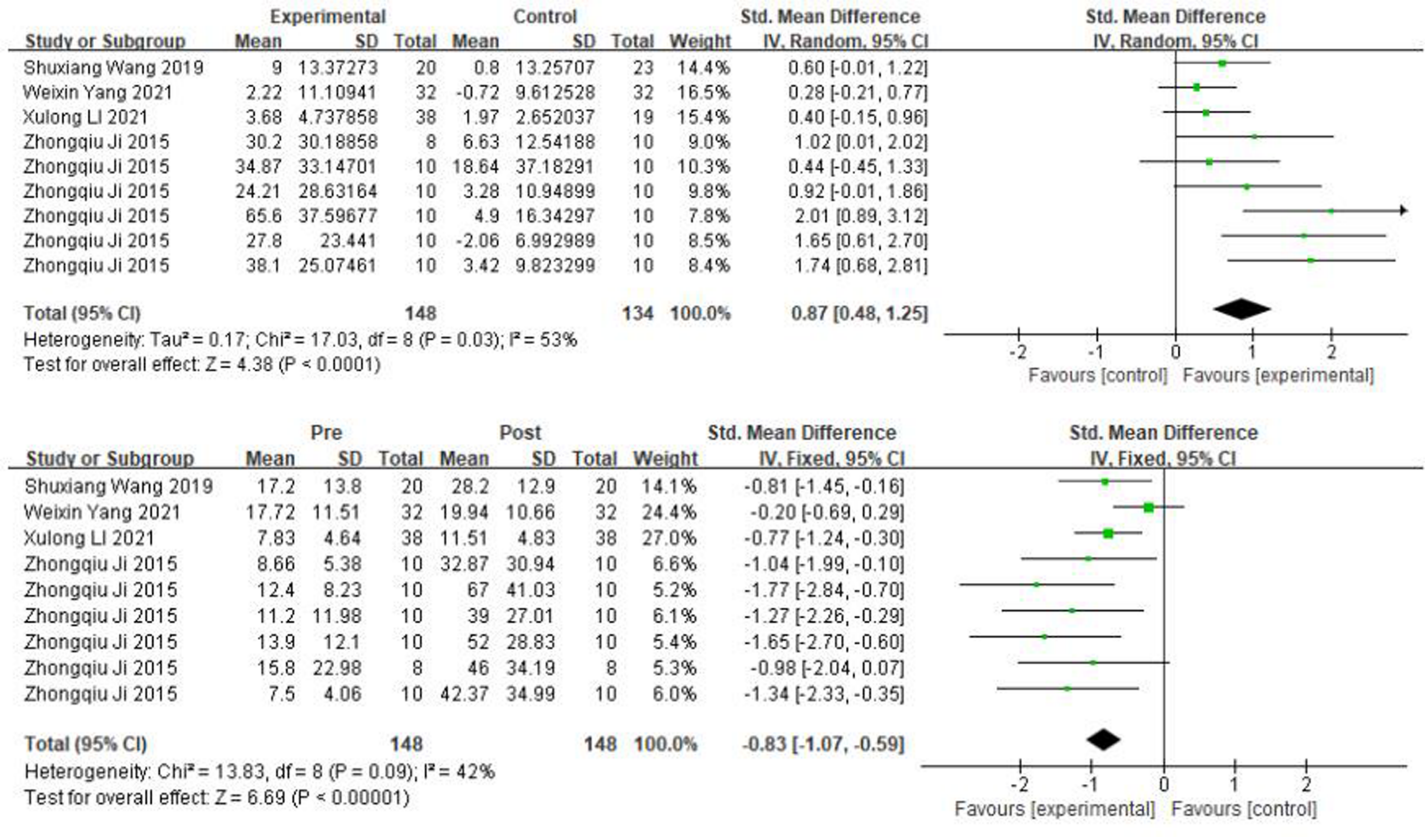
A meta-analysis of the results of closed-eye single-leg test was conducted for comparisons between the control and experimental groups and for comparisons between the experimental groups pre- and post-intervention.

In this study, a total of seven included studies were conducted on children's stabilization ability. The closed-eye single-leg test and flamingo balance were used as an index. From Figure 8, *χ*² = 17.03, df = 8 (*P* = 0.03), and *I*^2^ = 53% between the two groups of the closed-eye single-leg test, which can be considered as heterogeneity between the two groups, and therefore a random-effects model was performed. The results showed that the sample size was 282 cases, SMD = 0.87, 95% CI: [0.48, 1.25], *P* < 0.0001, and the diamond-shaped small squares were located to the right of the null line and did not intersect, indicating that there was a high effect and that the soccer practices had a significant contribution to the children's stabilization ability. Also we can see that pre- and post-intervention of the experimental group *χ*² = 13.83, df = 8 (*P* = 0.09), *I*^2^ = 43%, SMD = −0.83, 95% CI: [−1.07, −0.59], *P* < 0.00001, |SMD| > 0.8, the small diamond-shaped square was located to the left of the null line and did not intersect, it indicates that the level of standing on one foot with eyes closed was significantly higher in the experimental group after the intervention compared to before the intervention.

**Figure 9:**
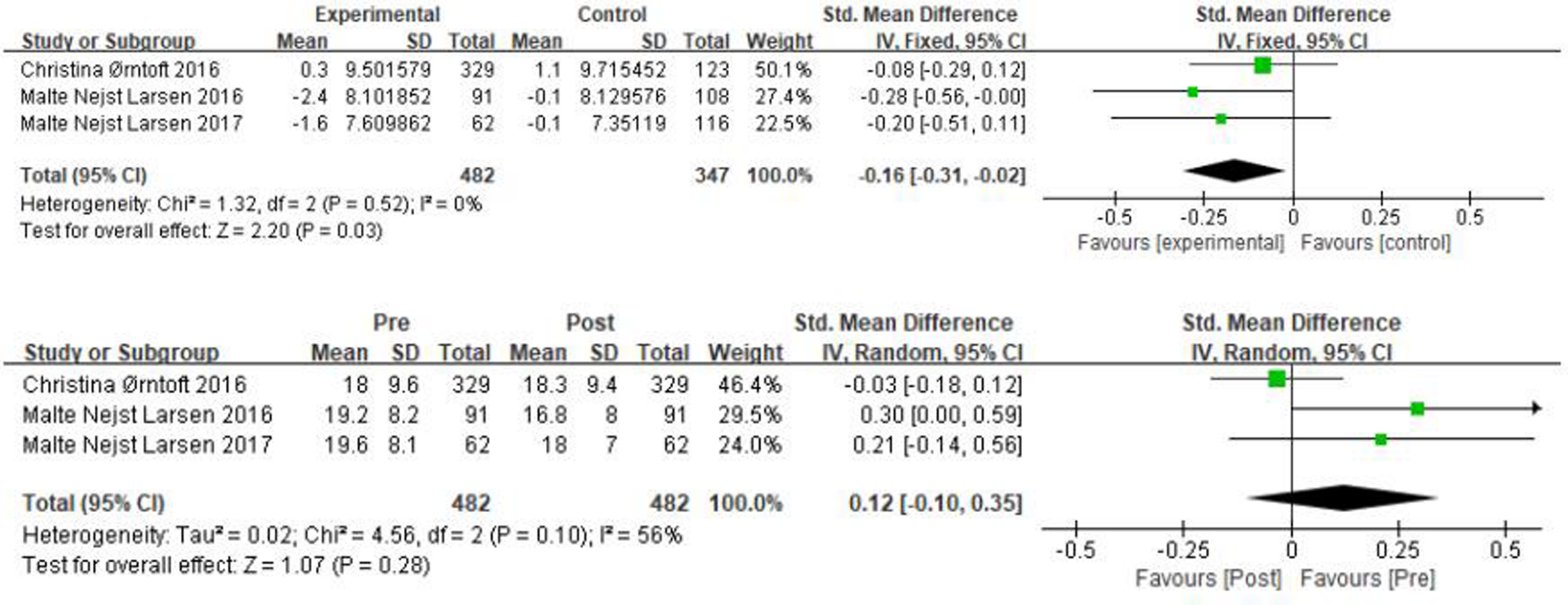
A meta-analysis of the results of flamingo balance was conducted for comparisons between the control and experimental groups and for comparisons between the experimental groups pre- and post-intervention.

In Figure 9, *χ*² = 1.32, df = 2 (*P* = 0.52), and *I*^2^ = 0% between the two groups of the flamingo balance test, which can be considered homogeneous between the two groups, and the fixed effects model was performed. The results showed that the sample size was 829 cases, SMD = −0.16, 95% CI: [−0.31, −0.02], *P* = 0.03, and the small diamond-shaped squares were located to the left of the null line and did not intersect, indicating that there was a small effect and that the soccer practices promoted children's stabilization ability. We can see that pre- and post- intervention in the experimental group *χ*² = 4.56, df = 2 (*P* = 0.1), *I*^2^ = 56%, SMD = 0.12, 95% CI: [−0.1, 0.35], *P* = 0.28 indicating that the difference in balance ability after the intervention in the experimental group was not statistically significant.

## Subgroup analysis

It was difficult to conduct subgroup analysis of intervention intensity and content because most of the included studies had the problem of not detailing the intensity of the intervention and the proportion of training content, while object control was not suitable for subgroup analysis because of the small number of included studies. Ultimately, this study subgroups three aspects of total intervention length, age (years), and gender for subgroup analysis.

### Comparison of the effects of different total intervention durations

In this study, the total intervention period (weeks), weekly intervention frequency, and length of each lesson(min) were considered to be factors influencing the study results, and subgroup analysis of one of them alone would result in the study outcomes being confounded by the other two factors. Therefore the “total intervention period (weeks)” × “weekly intervention frequency” × “length of each lesson(min)” was used to obtain the total intervention duration, which was used to explore the relationship between intervention time and effect. In terms of the degree of distinction as well as the rationality of the intervention, “15 weeks * 3 times/week * 40 min/time  =  1,800 min” was used as the dividing line. In Table 3, the|SMD_1,800 min below_| < |SMD_1,800 min more_| of linear sprint ability and horizontal jump, the intervention above 1,800 min was a moderate and high effect, while below 1,800 min was a small effect, so the training effect above 1,800 min promoted the development of linear sprint ability and horizontal jump more. Flamingo balance had no statistically significant difference between groups below 1,800 min, while there was a large effect above 1,800 min. The duration of the closed-eye single-leg test intervention was below 1,800 min, and there was a high effect. There was no statistically significant difference between groups above or below 1,800 min for the countermovement jump.

**Table 3:** Comparison of the effects of different total intervention times.

Test index	Subgroup	Number of articles	*Z*	*P*	SMD 95% CI
Linear sprint ability	1,800 min more	6	2.57	0.01	−0.43 [−0.76, −0.1]
	1,800 min below	3	1.64	0.1	−0.32 [−0.71, 0.06]
	Overall	9	3.11	0.002	−0.37 [−0.61, −0.14]
Horizontal jump	1,800 min more	2	2.96	0.003	1.16 [0.39, 1.93]
	1,800 min below	4	1.87	0.06	0.34 [−0.02, 0.7]
	Overall	6	2.92	0.003	0.62 [0.21, 1.04]
Countermovement jump	1,800 min more	2	0.85	0.4	0.78 [−1.02, 2.57]
	1,800 min below	1	0.77	0.44	0.22 [−0.34, 0.77]
	Overall	3	1.15	0.25	0.5 [−0.35, 1.35]
Closed-eye single-leg test	1,800 min more	0	–	–	–
	1,800 min below	4	4.38	<0.0001	0.87 [0.48, 1.25]
	Overall	4	4.38	<0.0001	0.87 [0.48, 1.25]
Flamingo balance	1,800 min more	2	2.32	0.02	−0.99 [−1.44, −0.55]
	1,800 min below	1	0.79	0.43	−0.08 [−0.29, 0.12]
	Overall	3	2.2	0.03	−0.16 [−0.31, −0.02]

### Comparison of effects by age group

In this study, the studies were divided into two subgroups based on age: 7–9 years old and 10–13 years old. In Table 4, there was no statistically significant difference between the groups of linear sprint ability and horizontal jump in children aged 10–13 years old, but there were medium and small effects in 7–9 years old, and it can be concluded that the effect of soccer practices on linear sprint ability and horizontal jump in children aged 7–9 years old was better than that in 10–13 years old, and there was no statistically significant difference between the groups of countermovement jump and flamingo balance in both 7–9 years old and 10–13 years old, while the eyes closed Closed-eye single-leg test SMD_7–9years_ < SMD_10–13years_, indicating that children aged 10–13 years had better intervention effects on the ability to stand on one leg with eyes closed.

**Table 4:** Comparison of the effect of different age groups.

Test index	Subgroup	Number of articles	*Z*	*P*	SMD 95% CI
Linear sprint ability	7–9 years old	4	2.74	0.006	−0.47 [−0.81, −0.13]
	10–13 years old	4	1.61	0.11	−0.3 [−0.66, 0.06]
	Overall	9	3.11	0.002	−0.37 [−0.61, −0.14]
Horizontal jump	7–9 years old	3	1.97	0.05	0.93 [0.01, 1.84]
	10–13 years old	3	1.73	0.08	0.35 [−0.05, 0.75]
	Overall	6	2.92	0.003	0.62 [0.21, 1.04]
Countermovement jump	7–9 years old	1	0.77	0.44	0.22 [−0.34, 0.77]
	10–13 years old	1	0.22	0.83	−0.08 [−0.82, 0.66]
	Overall	3	1.15	0.25	0.5 [−0.35, 1.35]
Closed-eye single-leg test	7–9 years old	4	3.15	0.002	0.46 [0.17, 0.74]
	10–13 years old	1	5.79	<0.00001	1.53 [1.01, 2.04]
	Overall	4	4.38	<0.0001	0.87 [0.48, 1.25]
Flamingo balance	7–9 years old	1	1.97	0.05	−0.28 [−0.56, 0]
	10–13 years old	2	1.37	0.17	−0.12 [−0.29, 0.05]
	Overall	3	2.2	0.03	−0.16 [−0.31, −0.02]

### Comparison of effects by gender group

The study was divided into three subgroups: “male”, “female”, and “mixed” according to the gender of the intervention subjects. In Table 5, linear sprint ability and closed-eye single-leg test |SMD _female_| >|SMD _male_| indicated that the soccer intervention was more effective for girls. There was a high effect of horizontal jump in boys, but no statistically significant effect in girls. There was no statistically significant effect for both subgroups of countermovement jump and flamingo balance.

**Table 5:** Comparison of effects by gender.

Test index	Subgroup	Number of articles	*Z*	*P*	SMD 95% CI
Linear sprint ability	Male	6	2	0.05	−0.49 [−0.98, −0.01]
	Female	3	2.21	0.03	−0.73 [−1.39, −0.08]
	Mixed	3	1.42	0.15	−0.11 [−0.27, 0.04]
	Overall	9	3.11	0.002	−0.37 [−0.61, −0.14]
Horizontal jump	Male	2	2.94	0.003	0.8 [0.27, 1.34]
	Female	1	1.54	0.12	0.81 [−0.22, 1.85]
	Mixed	3	1.26	0.21	0.23 [−0.13, 0.59]
	Overall	6	2.92	0.003	0.62 [0.21, 1.04]
Countermovement jump	Male	2	0.85	0.4	0.78 [−1.02, 2.57]
	Female	0	–	–	–
	Mixed	1	0.77	0.44	0.22 [−0.34, 0.77]
	Overall	3	1.15	0.25	0.5 [−0.35, 1.35]
Closed-eye single-leg test	Male	3	4.26	<0.0001	0.91 [0.49, 1.33]
	Female	1	2.65	0.008	1.35 [0.35, 2.35]
	Mixed	2	1.87	0.06	0.73 [−0.03, 1.49]
	Overall	4	4.38	<0.0001	0.87 [0.48, 1.25]
Flamingo balance	Male	1	1.27	0.2	−0.2 [−0.51, 0.11]
	Female	–	–	–	–
	Mixed	2	1.81	0.07	−0.15 [−0.32, 0.01]
	Overall	3	2.2	0.03	−0.16 [−0.31, −0.02]

## Discussion

### Soccer and children's locomotion

Locomotion is an indispensable ability in soccer, and linear sprint ability and jumping ability are the two main indicators to judge locomotion. The results of study have identified the facilitative effect of soccer practices on linear sprint ability, and there are relevant studies that have demonstrated this (17, 35, 36). It is related to the soccer itself including running, kicking, and shifting movements, but the findings of M.N. Larsen (15) in 2017 diverge from the findings of this study, whose intervention period was 43 weeks long and with an exercise intensity of 70%–90% HRmax, but the weekly intervention duration was only 60 min, which may indicate that long-period, short-per-intervention methods are not effective in improving linear sprint ability. Jianing He (36) showed a more significant improvement in linear sprint ability after 12 weeks of soccer technique and ball handling intervention at 270 min per week, which may indicate that a longer weekly intervention duration can better promote linear sprint ability improvement. A subgroup analysis showed that interventions longer than 1,800 min were more effective than those below 1,800 min, that interventions were more effective in children aged 7–9 years than in those aged 10–13 years, and that interventions were more effective in girls than in boys, suggesting that soccer interventions should be conducted at ages 7–9 years to improve linear sprint ability. Meanwhile, it is difficult to compare the effects of factors such as the ratio of physical fitness training to soccer technique training, the ratio of different soccer technique training, and the ratio of aerobic to anaerobic training on linear sprint ability in the intervention because subgroup analysis of the intervention content was not conducted in this study.

Secondly, the results of the study showed that the soccer practices had a facilitating effect on horizontal jump, and Yan Zheng (37) showed a significant improvement in children's jumping ability after a 32-week * 1 time/week * 120 min/time intervention, which might indicate that long-term low-frequency high-intensity prolonged soccer sports have a better effect on jumping ability, while C. Ørntoft (14) showed that after a 12-week * 2 times * 45 min intervention, the intervention effect in the experimental group did not differ from the control group, considering its similarity to the intervention content of Yan Zheng (37), we therefore hypothesized that a low frequency, short duration intervention would be ineffective. The subgroup analysis of the study also proved the important role of intervention duration and age on horizontal jump ability, as shown by the fact that the effect of intervention duration >1,800 min was better than that of <1,800 min, which also reminds us that the intervention duration should be extended in future interventions to improve the effect, while there was no significant difference between the intervention effect of boys and girls. Meanwhile, the effect of practice intensity on horizontal jump was studied (40), and in the medium- and high-intensity soccer practice, soccer players who received the high-intensity intervention performed significantly better in the horizontal jump than those with moderate practice intensity, this is consistent with Fei Xin's conclusion. For the countermovement jump, the results showed no difference between the experimental and control groups after the intervention, perhaps caused by the same participation of the control group in the physical education program, which is consistent with the findings of Xulong Li (19) and N. Cvetković (33), in addition to the findings of some of the studies (15, 41) that could not be combined due to different data presentation, which supports the conclusion. Whereas there were differences before and after the intervention in the experimental group, indicating that the soccer practices were effective for children, but the effect did not differ from participation in a general physical education program, the results of the countermovement jump should be treated with caution due to the small number of included studies.

### Soccer and children's object control

The present study found that soccer had a good promotion effect on children's object control in general, but there was no statistical significance between groups on stationary dribbling and catching. This is consistent with the conclusion reached by Yangyang Guo (13), in which there was a significant improvement in two-hand striking a stationary ball and throwing ability, and slightly higher scores on stationary dribbling and catching than the control group but no significant difference, he concluded that kicking movements involve precision control of the limbs on the target, and long-term practice tends to improve proprioception to help develop object control in the upper limbs. In contrast, the results of the study by P.F. Nazario (12) yielded the same results in terms of the promotion of stationary dribbling, but with a significant increase in catching ability, and it concluded that the characteristics of the experimental subjects, the environment they were in, and the time of the intervention were all influential factors. Considering the small difference between the two in terms of age and that the intervention by Yangyang Guo (13) was not traditional soccer instruction but ball practice, this study hypothesized that when the intervention included passing and catching football, upper limb throwing and catching accuracy would also be enhanced, but when the intervention was only ball practice and lacked passing and catching football, the enhancement of all upper limb object control abilities would be limited. Since this study included less study on upper limb object control, the specific effect still needs to be verified in the future. And since soccer itself is a sport involving lower limb control, most FMS assessment tools use soccer as a test of lower limb object control, so the effect of soccer on lower limb object control is direct, and this study used kicking in TGMD-2 as a test item, and the results showed that soccer had a facilitating effect on the effect of lower limb intervention, and not only that, Xulong Li (19) found that children's ability to change direction and dribble was improved after the soccer intervention, which can also represent the improvement of children's lower limb object control ability, although the specific intervention effect can be affected by various aspects such as training content, time, and characteristics of the experimental subjects, but the appropriate intervention has a positive effect.

### Soccer and children's balance

Balance is an important prerequisite for the development of motor skills, and examining children's balance will help future research and interventions that will lead to better overall movement skills (42). The results of the study point to differences in the results of the two different testing methods. In the four studies tested by Closed-eye single-leg test, the experimental group significantly improved compared to the control group, which is consistent with the study of Zhongqiu Ji (20), which concluded that movements such as running, paddling and dribbling have a helpful effect on stability, while the fatigue resistance of the calf muscles is also a major factor affecting balance, and soccer contains both aerobic and anaerobic exercises that promote fatigue resistance in the calves. And Xulong Li (19) and Shuxiang Wang (17) although there was no significant difference between the experimental and control groups, there was a significant difference between the posttest of the experimental group compared to the base test, as the balance ability can be effectively improved by practice can be improved (43), which may be related to the participation of the control group in the normal physical education program. The intervention effect was better in girls and 10–13 years old than in boys and 7–9 years old in the subgroup analysis, indicating that there are gender differences in FMS (44, 45) and it is more appropriate to intervene in soccer at the age of 10–13 years old in children, but the present findings do not coincide with the developmentally sensitive period of balance ability, and considering that the present study did not analyze factors such as practice content, ratio, and intensity, more research in this area is necessary in the future. However, the analysis results of the flamingo balance test showed that the balance ability was not improved significantly after the intervention, which can have an impact on the accuracy of the results due to the small amount of study, but it is worth noting that C. Ørntoft (32) in the present study performed an intervention with a total duration of 1,080 min, balance ability did not improve, while subgroup analysis, for >1,800 min study showed that the soccer practices had a better effect on the flamingo balance test, indicating that the increase in the total duration of the intervention was a major factor in improving the effect of the intervention. Secondly, related scholars (40) conducted flamingo balance tests for 11-year-old soccer players after medium- and high-intensity soccer interventions with regular children, and the results showed that the balance ability of soccer players was significantly better than that of regular children, and the balance ability of soccer players with high exercise intensity was better than that of athletes with medium intensity.

### Limitations and shortcomings

The search terms in this study only included Chinese and English, and some of the studies could not be viewed in full text, resulting in fewer included study for the countermovement jump, object control ability, and flamingo balance tests, which had some impact on the reliability of the results. In the quality evaluation of the included study, most of the study failed to accurately state the principle of random assignment, and in some experiments where the subjectivity of the raters was relatively high, the scores were not blinded, and some of the literature did not report the withdrawal, resulting in a lower level of quality of the study. In the subgroup analysis, the small number of studies for some indicators and the difficulty in unifying the intervention intensity and proportion of experimental subjects across the study resulted in the inability to further analyze the interventions.

## Conclusions and recommendations

The soccer practice was able to improve children's FMS, as evidenced by significant improvements in linear sprint ability [SMD 95% CI = −0.37 (−0.61,−0.14), *P* = 0.002], horizontal jump [SMD 95% CI = 0.22 (−0.34,0.77), *P* = 0.003], object control [SMD 95% CI = 1.32 (0.8,1.85), *P* = 0.0003], Closed-eye single-leg test [SMD 95% CI = 0.87(0.48,1.25), *P* < 0.0001], and no improvement in countermovement jump, basketball shooting, flamingo balance test and catching test. The accuracy of the results needs to be verified in the future because of the small amounts of included studies in countermovement jump, upper limb object control ability, and flamingo balance test. Meanwhile, the subgroup analysis revealed that the intervention effects of linear sprint ability, horizontal jump and flamingo balance test were better than those of <1,800 min; the intervention effects of linear sprint ability and horizontal jump were better than those of 10–13 years old for children aged 7–9 years old, while the intervention effects of Closed-eye single-leg test were better for 10–13 years old than those of 7–9 years old; the intervention effects of linear sprint ability and Closed-eye single-leg test were better for girls. but the horizontal jump is better for boys to improve the effect.

It is suggested that when developing children with FMS in the future, the intervention effect can be increased by increasing the total length of intervention and selecting the appropriate age for timely development. The effects of different intervention components and the ratio of each component as well as the intensity of the intervention on FMS are lacking in the current study and need to be further explored in the future.

## Data Availability

The original contributions presented in the study are included in the article/Supplementary Material, further inquiries can be directed to the corresponding author/s.
